# Time‐Dependent Voronoi Analysis of Amino Acid Side‐Chain Packing in Simulations

**DOI:** 10.1002/jcc.70499

**Published:** 2026-09-21

**Authors:** Sofiya B. Bettencourt, Wonmuk Hwang

**Affiliations:** ^1^ Department of Biomedical Engineering Texas A&M University College Station Texas USA; ^2^ Department of Materials Science & Engineering Texas A&M University College Station Texas USA; ^3^ Department of Physics & Astronomy Texas A&M University College Station Texas USA; ^4^ Center for AI and Natural Sciences Korea Institute for Advanced Study Seoul Republic of Korea

**Keywords:** molecular dynamics simulation, protein packing, Voronoi tessellation

## Abstract

Protein conformational motion depends on geometric and chemical constraints in side‐chain packing. Thermal forces and incomplete packing produce “breathing” motions that allow internal rearrangement. Packing dynamics therefore inform protein allostery, conformational change, and folding. While Voronoi tessellation is a promising approach to this end, its application to proteins has been mainly focused on static structures. Here, the Voro++ library is adopted to investigate time‐dependent amino acid packing in molecular dynamics trajectories of a titin immunoglobulin domain at 300 and 400 K. Tracking per‐residue Voronoi volume changes and reorganization of neighbor faces in the Voronoi lattice reveals residue‐level volume shifts and the corresponding timing of localized protein swelling. Side‐chain motion into and out of the protein core can also be monitored by mapping neighbor switching. This framework is applicable to detecting delicate changes preceding protein conformational transition or unfolding, and analyzing protein–protein interface reorganization or surface hydration.

## Graphical abstract

Protein volume fluctuation is largely driven by loosely packed amino acid side chains while the backbone is comparatively restrained. We present a Voronoi‐based framework for quantifying and tracking local side‐chain volume and reorganization of neighbors. Paired snapshots show Voronoi tessellations for a side chain during molecular dynamics simulation that changes volume over time.

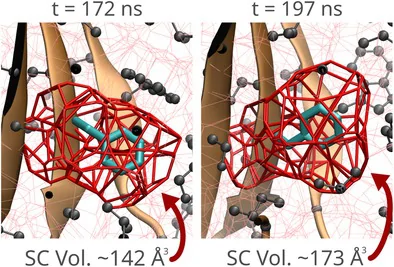

## 1 Introduction

Protein packing has long been a subject of significant interest [1, 2], concerning topics including protein–water interface and protein folding [3, 4, 5]. A related topic of protein allostery has yielded the development of numerous computational methods to understand information transfer across a protein [6, 7, 8, 9, 10, 11]. Many of them focus on the protein backbone motion rather than alterations in side‐chain packing as a significant component of allostery mechanism. For example, backbone heavy atom or C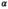 atom‐based measurements such as root‐mean‐square fluctuation (RMSF), radius of gyration, and C
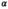
‐based contact maps are often used to infer packing or stability of a protein core. However, analyzing side‐chain dynamics is essential as they determine the chemical specificity. Side chains also generally take up more than half of a protein volume [3, 12, 13].

In molecular dynamics (MD) simulation, allostery can be probed through correlation analysis [14], or via analysis of mechanical strain [15, 16, 17], and stress [18]. Such analyses rely on identifying residues in close proximity that are often determined by using an arbitrarily defined cutoff distance based on positions of C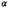 atoms [14] or by using more physically defined contacts such as hydrogen bonds or van der Waals (VDW) contacts [19, 20]. The cutoff‐based method may count residues that are unimportant in relaying information as neighbors. Conversely, it may leave out residues that are located beyond the cutoff distance but are crucial for information transfer. Approaches based on physical contacts suffer less from arbitrarily defined cutoff distance since the hydrogen bonding distance and VDW radii of atoms are well established. But the resulting network of atomic‐level contacts is complex to analyze from the point of view of packing which is a more coarse grained information that does not have one‐to‐one correspondence with precise arrangement of contacts.

Complementary information can be gained from Voronoi tessellation [21] that is used to study the geometry of point clouds in space [22]. Each point is assigned a unique Voronoi cell that encompasses the region of the domain closer to that point than any other. In a 3‐dimensional system, the face of a Voronoi polyhedron forms a perpendicular bisecting plane between two adjacent points. When applied to atoms or groups of atoms of a protein, Voronoi tessellation provides a metric for packing [2, 12]. Accordingly, various Voronoi‐based software tools have been developed to analyze, for example, cavity formation [23, 24], voids in folded proteins [25], internal volumes [12], protein channels [26, 27, 28, 29], and identifying regions of imperfect packing in a protein core [30, 31, 32, 33]. Most of these tools focus on analyzing static structures. While some can be iterated over simulation trajectories [34, 35, 36], their application to dynamic aspects such as position‐ and time‐dependent changes in residue volumes have not been extensively explored.

To apply Voronoi tessellation to simulation trajectories and probe intra‐protein dynamics and rearrangement, a low‐level control of Voronoi cell outputs and statistics is desirable. To this end, we develop a modular tool written in C++ that builds and analyzes Voronoi tessellation across coordinate frames. For speed and versatile output functionality, we adapt the widely used Voro++ library primarily designed for applications in physics and materials science [37, 38]. We create a postprocessing pipeline to map the Voronoi cell outputs into per‐residue data, allowing for selective analysis of either side‐chain dynamics or the full protein including the backbone. This approach thereby enables probing subtle breathing (local volume fluctuation) as well as reorganization of the protein interior during a simulation.

As a demonstration, we use this method to compare two MD simulations of the titin I27 domain [39] at 300 and 400 K. We characterize the reorganization of the protein domain via volume fluctuation and shifts in Voronoi polyhedra face neighbors. This enables pinpointing localized changes over time and the corresponding order of residue‐level reorganization. Our Voronoi‐based analysis can further utilize metrics such as residue neighbor statistics, residue volumes, and side‐chain polyhedra geometries. We thus expect it to be useful for extracting geometric yet physically relevant information from atomistic simulation trajectories.

## 2 Methodology

### 2.1 Preprocessing and Voronoi Tessellation

**FIGURE 1:**
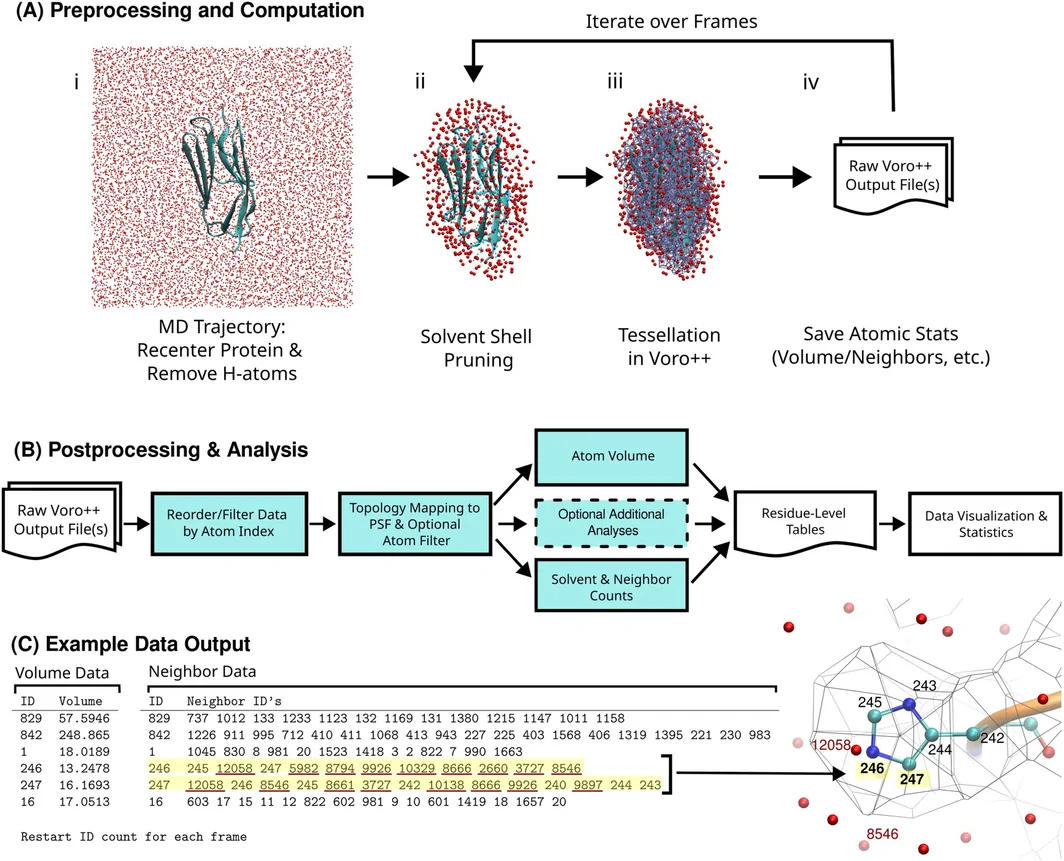
Method overview. (A) Preprocessing and computation of Voronoi polyhedra. (B) Postprocessing and analysis pipeline. (C) Example volume and neighbor data around His31. In highlighted lines, indices corresponding to solvent are underlined. The tessellation for water (red spheres) was not visualized and some neighbors were cropped for clarity.

Our main workflow is summarized in Figure 1. We preprocess the CHARMM [40] DCD format coordinate trajectory files where the protein is aligned to a reference structure (the first frame was used). This is done by using the CHARMM MERGe command in two steps. First, the simulation box is translated in a periodic sense so that it diffuses with the protein located at the center of the box. Next, each frame is aligned with respect to the protein at the first frame. Since the solvent box moves with the protein, aligning to the first frame results in the entire simulation box rotating about the protein while removing rotational diffusion of the protein. Due to the minimum distance of 12 Å added between the protein and the boundary of the solvation box during the system preparation, the above procedure ensures the distance from the protein to the boundary of the solvation box sufficiently greater than 5 Å that was included in the Voronoi analysis (Figure 1A‐i).

All hydrogen atoms are removed to reduce the system size, and we iterate over each frame to keep only the solvent (including ions) within 5 Å of the protein heavy atoms (7 Å for the 400 K system to accommodate overall increased volume). The retained solvent molecules were re‐indexed with contiguous integers counting from 1 by using the RENUmber command of CHARMM. Linear spans of the system along three Cartesian coordinate axes (box dimension after solvent shell pruning; obtained via the CHARMM COORdinate STATistics command) were also recorded for each frame (Figure 1A‐ii). The resulting solvent layer provides a boundary where Voronoi cells of surface residues in contact with solvent can be built. All CHARMM scripts used to perform the preprocessing of simulation trajectories are in the GitHub repository linked in the Code Availability section.

The coordinates and the box dimension were used to compute the tessellation via the Voro++ library (Figure 1A‐iii). To allow quick identification of neighbor atoms during Voronoi cell construction, the system was subdivided into a 6×6×6 rectangular grid where there were 5–8 atoms per cell on average [38]. The grid size is an input parameter for Voro++ that can be adjusted depending on the system size. The output data for individual frames were written into a file for subsequent analysis.

### 2.2 Postprocessing of Voronoi Data

The raw Voro++ output includes unordered lists of atom indices and corresponding geometric statistics (e.g., volumes, neighbor lists, and vertex positions). We order them by heavy‐atom index matching the protein structure file (PSF) atom order (box 2 in Figure 1B). Example raw data outputs are shown in Figure 1C. Atoms with ID's 246 and 247 are visualized along with some protein and solvent (red dot) neighbors. The lists are trimmed to only include protein atoms, removing any data for solvent atoms that have indices greater than the total protein atom count. This excludes additional analysis of solvent itself while information about solvent neighbors for protein atoms are retained in the Voro++ output (Figure 1C, underlined indices). The PSF for the simulation system is used to generate a lookup table that maps atom indices to protein residues. In this step, the user can isolate specific components of the system, such as backbone atoms or protein side chains (third box in Figure 1B).

C++ functions were written to handle different types of data in output files. For volume analysis, heavy atom volumes were summed for each residue, with an option for backbone exclusion. For neighbor analysis, the ordering of indices, where solvent atoms appear after protein atoms was utilized to distinguish Voronoi cell interfaces of each residue formed with other residues versus with solvent molecules (Figure 1C, underlined indices). When counting neighbors, multiple interfaces for atoms belonging to a single neighboring residue were combined to count only as one neighbor. An illustration of this is shown in Figure 1C, where both atoms 246 and 247 of His31 share solvent neighbors with IDs 12058 and 8546. In the postprocessing, each solvent neighbor is counted only once for this residue. The final result is two separate datasets; one for residue‐residue contacts and another for residue‐solvent contacts.

### 2.3 Analysis, Plotting, and Visualization

We used the NumPy module of Python for statistical analysis and Matplotlib or Pandas for plotting data. In addition to using Voro++ for calculating volumes of residues, we used the CHARMM COORdinate VOLUme command for comparison. Visualization of molecular structure and Voronoi cells were done by using VMD [41] by converting and loading the vertex and edge data of Voronoi cells as protein data bank (PDB) and CHARMM PSF format files.

### 2.4 MD Simulation

For MD simulation, we used a crystal structure of the titin I27 domain (PDB 1WAA; 1.80‐Å resolution) [39]. The CHARMM param36 all‐atom force field [42] was used. We used CHARMM [40] for system building, solvation, neutralization, and equilibration, and OpenMM [43] for production runs. The crystallographic unit of PDB 1WAA contains six chains, of which we used chain F which had no missing residues or atoms. We kept residues 1 through 89, and did not include the dangling N‐terminal 3‐aa strand that has residue ID range −2 to 0. Protonation states of histidines were assigned to promote hydrogen bond formation with atoms in nearby residues. This yielded protonation of N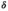 atom for His20 and His31, and protonation of N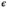 atom for His56 and His61. Crystallographic waters where the water oxygen is within 5 Å of the protein heavy atom were retained.

Upon building the structure, a quick 200‐step steepest‐descent energy minimization was performed, while protein backbone heavy atoms were positionally fixed. We oriented the protein so that the center of mass of C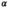 atoms is at the coordinate origin and the major axis is along the x‐axis by using the COORdinate ORIEnt command of CHARMM. The system was placed in a cubic water box where the box size was determined to have a minimum distance of 12 Å from the protein (including hydrogen atoms) to the boundary. We used the TIP3P water model and removed any water molecules with oxygen atoms less than 2.8 Å away from protein heavy atoms. The solvated system was neutralized by using Na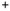 and Cl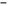 atoms at about 50‐mM concentration. The neutralized system was 75×75×75 Å^3^ in size, containing 41,700 atoms.

We performed a five‐stage energy minimization to gradually relax the solvent and protein atoms, as described previously [44]. The system was then heated from 30 to 300 K for 100 ps and equilibrated at 300 K for 200 ps. The 400‐K system was heated again after equilibration at 300 K, to 400 K for 100 ps, and equilibrated at 400 K for another 200 ps. All backbone heavy atoms were positionally restrained with 10‐kcal/[mol·Å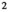] and 5‐kcal/[mol·Å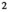] harmonic spring constant during heating and equilibration, respectively. Following equilibration, a 2‐ns constant pressure and temperature (CPT) simulation under 1 atm and at 300 or 400 K was performed. During this run, we applied a small 0.001‐kcal/[mol·Å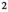] restraint on all C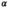 atoms of the protein. We used the DOMDEC [45] module of CHARMM for parallel computation in a computer cluster. We applied the SHAKE method to fix the hydrogen atom covalent bond length and used a 2‐fs integration time step. We used 12‐Å cutoff length for nonbonded interactions. The particle mesh Ewald (PME) summation algorithm [46] was used to account for long‐range electrostatic interactions.

Production runs were performed using OpenMM [43] for 200 ns at each of the two temperatures. We used a 12‐Å cutoff distance and an Ewald error tolerance of $10^{-4}$ for improved accuracy of the OpenMM run. The Nose–Hoover constant temperature integrator [47, 48] was used, with a 2‐fs integration time step. The simulations were run on NVIDIA A100 GPUs with mixed floating point precision. Coordinates were saved every 20 ps, yielding 10,000 frames for each 200‐ns simulation. For analysis, different intervals of the simulation were used to examine either the stable portion of the trajectory or local changes in side‐chain packing.

## 3 Results

### 3.1 Temperature‐Dependent Behaviors of the Total Voronoi Volume

**FIGURE 2:**
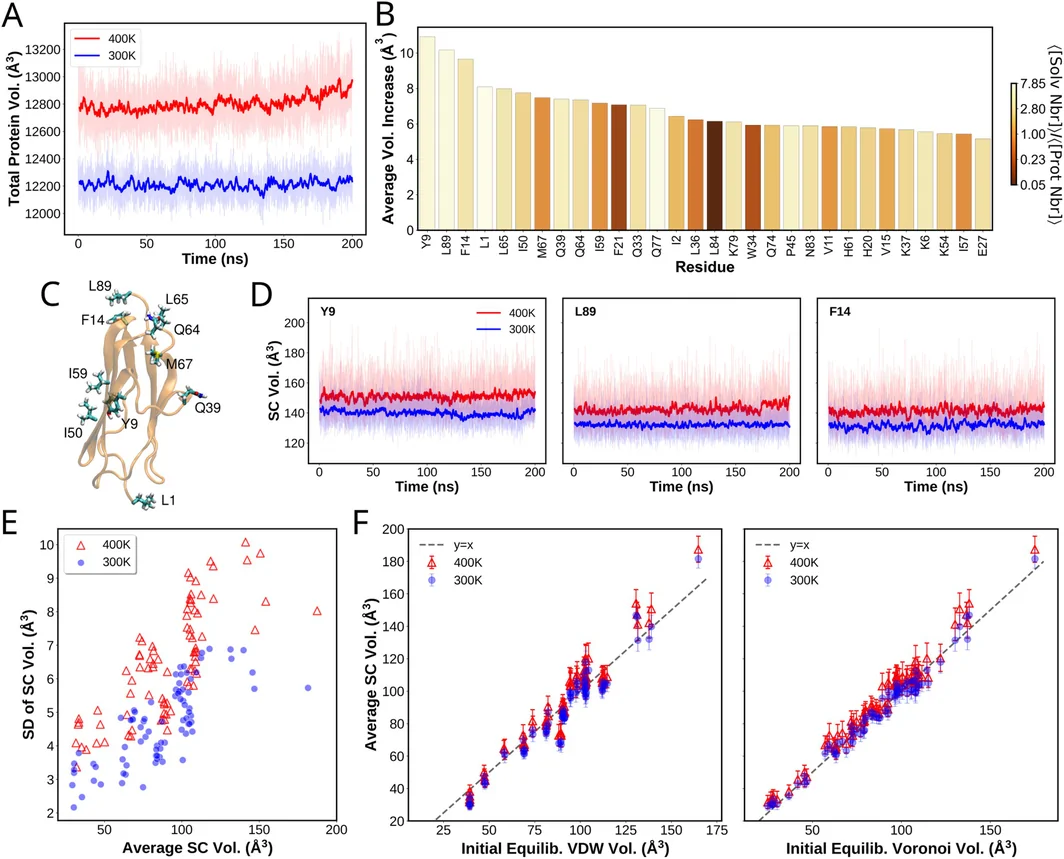
Time resolved residue‐level volume statistics for 300 and 400 K simulations. (A) Total protein volume over time. Thick lines: rolling average with a smoothing window of 50 frames. (B) Top 30 expanded side chains. Differences were taken with $\left\langle V_{i}^{400\;\text{K}}\rangle -\langle V_{i}^{300\;\text{K}}\right\rangle$ (*i*: residue ID). Color scale denotes the ratio of the average number of solvent neighbors versus the average number of protein neighbors as a measure of solvent exposure in the 300 K simulation. Note colors are in logarithmic scale. (C) Titin domain in ribbon representation with top 10 expanded side chains in stick representation. (D) Example time traces of top three expanded side‐chain volumes. Similar plots for all residues are in Figure S4. (E) Side‐chain volume fluctuation versus the average volume. (F) Comparison between the average side‐chain Voronoi volume during the simulation (*y*‐axis) and the VDW (left plot) or Voronoi (right plot) volumes of the initial equilibrated structure at 300 K. Averages in panels B, E, F are over the first 180 ns.

At 300 K, the protein remained stable as the total Voronoi volume for protein heavy atoms did not increase over time (blue line in Figure 2A). The stability can also be seen by the low root mean square deviation (RMSD) of backbone C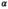 atoms from the first frame as well as the low RMSF throughout the simulation (blue lines in Figure S1A,B). Volumes of the protein and individual residues, whether buried or at the surface, remained stable during the 300‐K simulation. At 400 K, the total volume was greater and it gradually increased over time (red line in Figure 2A). The difference in the total Voronoi volume between 300 and 400 K was 624 Å^3^ on average (average was over all 200 ns). The difference at 400 K can also be seen by the corresponding RMSD and RMSF profiles (red lines in Figure S1A,B). The volume increase over time at 400 K is mainly due to changes in the loop motion and internal packing shifts, as explained below.

In comparison, the total Voronoi volume of the protein backbone was 5127 Å^3^ at 300 K which is about 42% of the total Voronoi volume of the whole protein (12,211 Å^3^) (Figure 2A vs. Figure S2). At 400 K, the total Voronoi volume of the backbone was on average 204‐Å^3^ larger than the value at 300 K (Figure S2). This is about 3‐fold smaller than the 624‐Å^3^ difference for the whole protein at the two temperatures. Thus, side chains are mainly responsible for the increase in volume at 400 K.

### 3.2 Residue‐Level Properties of Voronoi Volumes

We considered volumes of individual side chains by excluding Voronoi cells corresponding to the protein backbone. The increase in side‐chain volume at 400 K was generally greater for surface residues compared to buried ones. This can be seen from the list of the most expanded side chains where top‐ranked ones were mostly exposed residues, although not all surface‐exposed residues experienced a large volume increase (Figure 2B,C; see the color scale in panel B). Traces for the top three residues that experienced the greatest volume difference between 300 and 400 K show increased volume throughout the simulation (Y9, L89, and F14; Figure 2D). Their increased volumes are thus mainly due to greater thermal fluctuation at the elevated temperature rather than due to conformational change occurring during the production run. For L89, the last residue of the protein, there is a slight increase in side‐chain volume after ∼175 ns (Figure 2D). This is due to an increased motion as L89 is not buried (Figure 2C). Its high solvent exposure is also seen in Figure 2B.

Figure S3A shows average side‐chain volume difference between 300 and 400 K plotted against the ratio of the average number of solvent neighbors versus protein neighbors for each residue. Although residues with a greater number of solvent neighbors generally experience a higher average volume change, the correlation is not strong (Figure S3A). Aside from the ratio of solvent versus protein neighbor counts being a simplistic measure of solvent exposure, the weak correlation is because in the small titin system, even the interior residues retain at least one solvent neighbor throughout the trajectory, which makes it difficult to establish any definitive correlation between solvent exposure and side‐chain volume expansion.

The average and standard deviation (SD) of side‐chain volume show overall positive correlation between the two where the 400‐K simulation shows a steeper increase in SD with average side‐chain volume (Figure 2E). To determine whether surface residues exhibit higher volume fluctuations compared to buried ones, we color‐coded this same plot by the log of the ratio of the average numbers of solvent neighbors to protein neighbors (Figure S3B), which does not show any clear trend. To further examine diversity in temperature‐dependent side‐chain volume expansion, we plotted trajectories of side‐chain volumes at 300 and at 400 K at for all residues (Figure S4). Some residues have higher and stable side‐chain volume from the start of the simulation at 400 K (e.g., Y9, F14, and E48). On the other hand, the side‐chain volume of I23 is nearly the same at the two temperatures, but later it rapidly increases at 400 K (Figure S4). The amplitude of the fluctuation in side‐chain volume also varies among amino acids (Figure 2E and Figure S4). Note that, such information is difficult to obtain from simple RMSD or RMSF measurements.

To examine the volume difference relative to the crystallographic state, we calculated side‐chain volumes after equilibration at 300 K. Since, the backbone heavy atoms are positionally restrained up to the equilibration phase, the backbone conformation is very close to the original crystal structure while exposed side chains equilibrate with the surrounding water molecules, which is needed to make Voronoi cells of surface‐exposed residues to reflect the solvated environment. For this structure, we used two different volume calculation methods, either based on VDW radii of the CHARMM param36 force field (for this, the COOR VOLUme command of CHARMM was used) or by measuring their Voronoi volumes. They were compared with the average side‐chain Voronoi volumes during the first 180 ns of simulation (Figure 2F). At 400 K, 21 out of 89 side chains had the average Voronoi volume during the simulation greater than the VDW volume of the initial structure by more than 1 SD, which is due to increased thermal motion. In contrast, at 300 K, average side‐chain Voronoi volumes of 40 out of 89 residues were lower compared to the initial structure by more than 1 SD, suggesting enhanced packing of side chains during simulation compared to the initial state. As shown previously [3, 12, 13], exposed side chains have larger Voronoi volumes, whereas residues in the protein core are more densely packed. Accordingly, deviations from the VDW volumes reflect whether a residue is solvent‐accessible or buried.

### 3.3 Tracking Side‐Chain Reorganization

**FIGURE 3:**
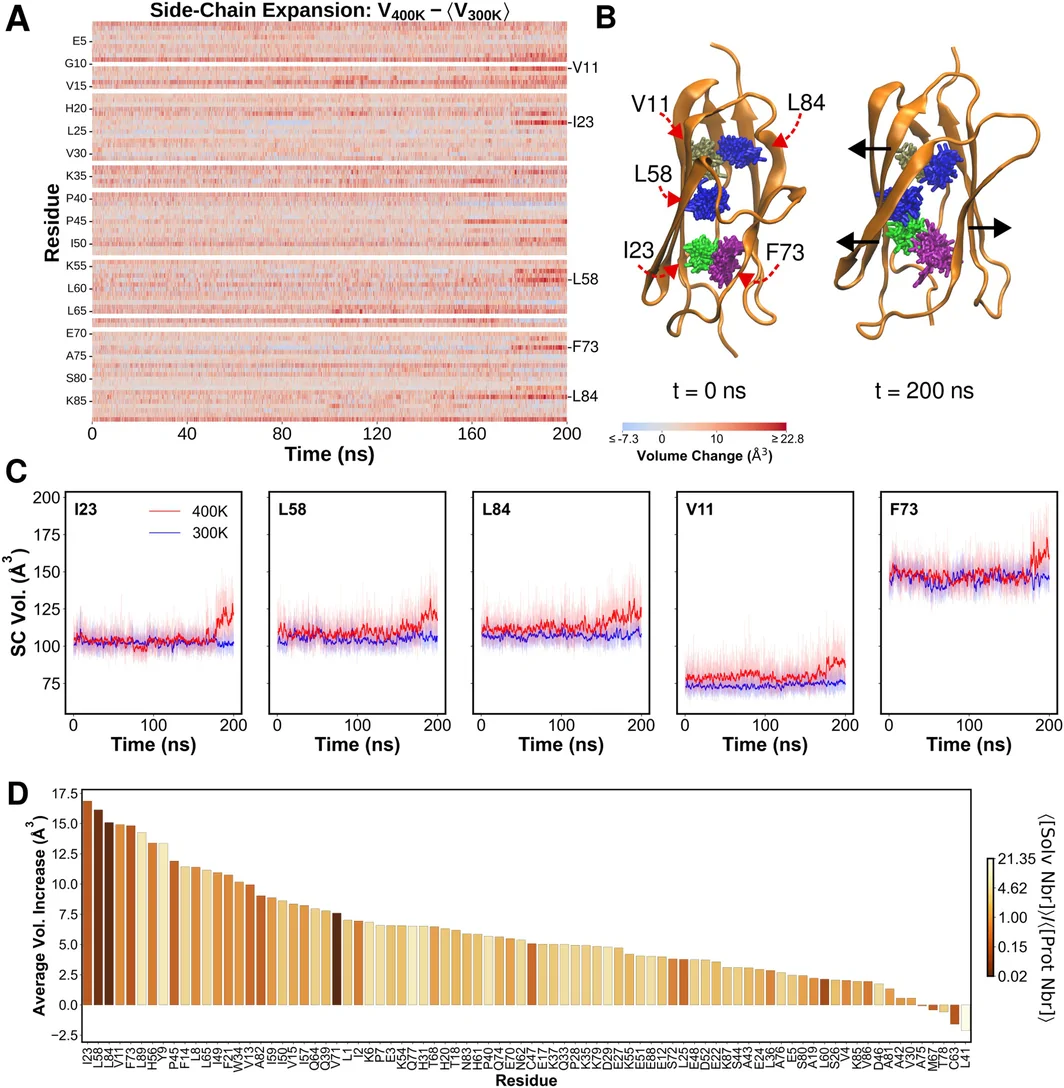
Intra‐domain side‐chain reorganization revealed by volume changes. (A) Time‐resolved heat map of side‐chain expansion. Volume change of a given residue at 400 K is measured relative to the average Voronoi volume at 300 K over the full trajectory. For visual clarity, the top and bottom 2% of data values are set to the maximum and minimum values of the color scale. Glycines have no side chain so the corresponding trajectories form white stripes. (B) Location/dynamics of the top five expanded side chains during the last 20 ns of simulation. Ensembles of 10 frames each from the initial (0–20 ns) and final (180–200 ns) simulation segments are overlaid on the starting and ending structure, respectively. Greater space covered by side chains in stick representation highlights increased local flexibility during the last 20 ns. Expansion of the β‐sandwich is indicated by black arrows. (C) Time traces of the residues from panel B. See Figure S4 for comparison with all other residues. For comparison, trajectories of backbone volumes for individual residues are in Figure S5. (D) Side chains ranked from the most expanded to the most contracted over the last 20 ns. Color scale is logarithmic as in Figure 2B.

Due to its stability, we used the average Voronoi volume of each side chain during the entire trajectory at 300 K as the reference for measuring time dependent changes in the side‐chain volume at 400 K (Figure 3A). Volume changes are prominent around 180 ns when the distance between the two β‐sheets increases, allowing internal side chains to reorganize (Figure 3B; Movie S1). This event is not highly distinguishable in the RMSF or RMSD data (Figure S1A,B) because an earlier event at 150 ns as explained in the neighbor analysis below impacts both metrics; the resulting sharp RMSD jump and localized RMSF increase obscure the β‐sandwich opening discussed here. For a given RMSD, since many different conformations are possible, once significant deviation occurs from the reference structure after 150 ns (Figure S1A), subsequent changes are not captured by RMSD. While it is to some extent possible to find the order of local changes by calculating RMSF in time intervals, this does not provide the time resolution and quantitative details at the level of individual amino acids as revealed by the Voronoi analysis. Furthermore, the increase in side‐chain volume across many residues as indicated by the appearance of red bands after ∼100 ns in Figure 3A elucidates events occurring well before the onset of any salient changes.

The early changes also manifest as a gradual increase in total protein volume (Figure 2A) and the RMSD (Figure S1A), while the corresponding backbone volume trace does not reflect the change (Figure S2). Examining local volume changes for individual residues (side chains in Figure S4 and backbone in Figure S5) shows that, although both side‐chain and backbone volumes at 400 K can change over time, sharp increase occurs only in side‐chain volumes of select residues. Such local changes may not be captured by the total volume change since the latter is a cumulative effect of side‐chain packing rearrangements and motion (Figure 2A). However, a clear difference in the total volume between 300 and 400 K (Figure 2A) indicates an overall difference in packing at the two temperatures even before any major conformational change occurs, which is not as clearly captured by the RMSD profile (Figure S1A).

Residues with highest average volume increase during the last 20 ns inform the internal reorganization event (Figure 3B). The top five expanded residues are all initially buried. Their individual time traces reveal the timing of their responses (Figure 3C). Notably, L84 (Figure 3A–C) exhibits an earlier increase in side‐chain volume whereas the remaining residues show a rapid increase during the actual reorganization event. On the other hand, some residues exhibit decrease in volume at 400 K compared to 300 K (blue bands in Figure 3A and negative values in Figure 3D). This is due to becoming more buried during the reorganization (T78 and A75) or due to loop motion (C63, M67, and L41) discussed in the following section.

### 3.4 Neighbor Analysis

**FIGURE 4:**
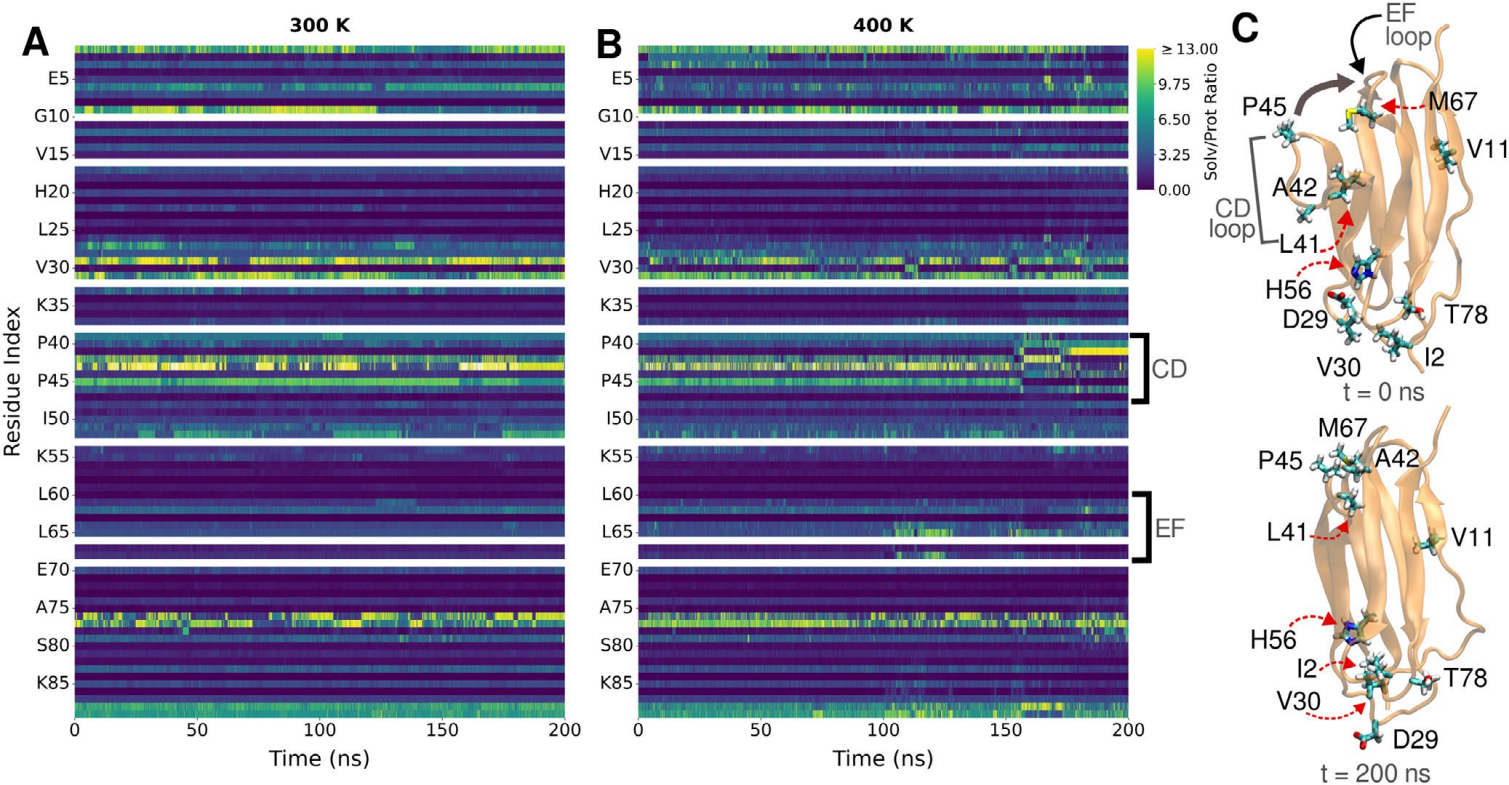
Switching between solvent and protein neighbors due to side‐chain motion in and out of protein core. (A, B) Instantaneous ratios between the numbers of solvent neighbors and protein neighbors for each residue at (A) 300 and (B) 400 K. CD and EF loops are marked. Some highly solvent‐exposed residues occasionally have zero protein neighbors making the solvent to protein ratio undefined. Such frames are colored white, though barely visible in the heat map due to their sparsity. In all other cases, the solvent‐to‐protein ratio falls within the range as indicated in the color bar, which is truncated at the 98th percentile to exclude outliers and enhance contrast (indicated by “≥13.00”) in the color scale. Glycines have no side chain and their trajectories form white stripes. (C) Snapshots of first and last frame (400 K) with top 10 residues by SD of protein neighbor counts shown in stick representation. Gray arrow shows loop movement at 155 ns. Also see Movie S1.

To gain insight into changes in solvent exposure over time, we took the ratio of solvent neighbors and protein neighbors at each time step (Figure 4A). A caveat of this ratio is that the number of neighboring Voronoi cells depends on the size of the side chains. Thus, it is difficult to establish a universal cutoff value for the ratio in determining whether a given side chain is solvent exposed or buried. While a more faithful approach may consider areas of Voronoi faces that neighbor solvent versus other protein residues, our simple measure of counting neighbors is effective for finding relative changes in solvent exposure for individual residues.

We rank‐ordered residues according to the average count‐based solvent exposure ratio (Figure S6). Compared to 300 K, at 400 K, more residues have the ratio greater than 1, with the value overall higher, as expected. When the ratios are traced over time for each residue, the simulation at 400 K exhibited significantly more fluctuation and shifts in solvent versus protein neighbors (Figure 4A,B). The heat map also captures the large‐scale shift in neighborhood assignments. At 155 ns, the CD loop (L41–P45) unbent and began to interact with its neighboring EF loop (Q64–T68; Figure 4C and Movie S1). This event also manifests as an increase in overall RMSD (Figure S1A) and the RMSF profile of the CD loop (Figure S1B). Unbending of the CD loop is accompanied by exchanges between solvent neighbors and protein neighbors, resulting in solvent exposure of L41 while other residues in the CD loop became more buried. Concomitantly, residues of the EF loop became slightly more buried, as can be seen by the color changes around 150 ns in the heat map (Figure 4B).

## 4 Concluding Discussion

We have demonstrated a Voronoi polyhedra‐based framework that identifies protein volume changes through residue‐level time traces. By leveraging neighbor data from Voronoi cells, we also reveal the dynamics of buried and exposed side chains, pinpointing specific areas of subtle rearrangements within the core packing. This pipeline can be further extended to track other Voronoi cell metrics such as face areas and identities of shifting neighbors.

While our comparative simulations at 300 and 400 K were relatively short, our approach would be effective for analyzing longer‐time simulations of larger proteins. For such systems, subtle internal shifts may be difficult to identify by other methods based on atomistic configurations such as contacts or dihedral angles where the relation to side‐chain packing is not straightforward. By comparison, since Voronoi tessellation is geometry based and depends less on atomistic details, it can inform more directly about packing. Specifically, we are able to see the rich diversity of responses to temperature increase among the side chains of the protein interior and exterior. Our approach thereby enables identifying regions of the protein experiencing “breathing” (increase or decrease in volume fluctuation) [49, 50, 51] and neighbor changes that indicate side chains moving in and out of the protein core or switching places with one another. We also demonstrate the ability to pinpoint changes and identify areas where intra‐protein shifts and “proteinquakes” [52] originate. Because of the stochastic nature of the simulation, the specific order of events of side chain shifts may depend on individual trajectories. In such case, multiple trajectories may be needed to show residues that consistently initiate changes before salient shifts occur in a protein.

Earlier studies applied Voronoi analysis to crystal structures of proteins to define residue volumes or solvent‐neighboring volumes [3, 12]. Since then, VDW radii have been established in biomolecular force fields [42], though they alone cannot inform the extent of protein packing. However, overall agreement between the Voronoi volume averaged during the simulation and the VDW volume indicates that the titin domain is well‐packed at 300 K (Figure 2F).

While Voronoi tessellation weighted by atomic radii may provide a more faithful residue volume estimation [13], for examining relative volumetric fluctuation rather than absolute individual volumes of side chains, weighted tessellation is not needed. Besides, shifting the dividing planes between atoms proportional to their atomic radii introduces vertex error [1, 2]. Likewise, the inclusion of hydrogens is not essential for detecting relative changes. Despite its simplicity, our approach captures delicate events such as the increase in the volume of L84 leading to more salient side‐chain rearrangement in the protein core (Figure 3A). If necessary, it is straightforward to include weighing by atomic radii through the “container_poly” feature available in the Voro++ library.

In addition to Voro++, there are other software that perform Voronoi analysis, including MOLE [27], TRAVIS [34], Voronoia [31], and Voronota [30]. While they have been used to track individual residue volumes over time [36, 53] or carry out neighbor detection [34], they are mainly focused on single quantities, primarily volume, or they include extra biologically‐relevant overhead such as categorizing output by residue type at each time step or higher‐level interfaces that limit modularity and make customized trajectory analysis less convenient. While it is possible to use those tools to extract more detailed Voronoi data, we chose Voro++ as there is little overhead regarding the system type or molecular selection, and since it enables a low‐level control where various quantities involving Voronoi cells can be directly extracted and analyzed.

The focus of many time‐resolved Voronoi analysis studies have been cavity identification and tracking [28, 35, 54]. Rather than looking for isolated voids within a protein with thresholds that determine when a cavity is formed, the focus of the present study is on the spatially resolved dynamics of volumes and neighbors across the protein as it undergoes thermal fluctuation or conformational change. The present Voronoi‐based approach can be further combined with other analysis methods such as contact network [19] or side‐chain conformational entropy [55], and surface hydration [56] for comprehensive analysis of protein dynamics and allostery.

## Funding

This work was supported by U.S. National Institutes of Health Grant No. P01AI143565.

## Conflicts of Interest

The authors declare no conflicts of interest.

## Supporting information




**Figure S1:** Conformational fluctuation during 300 and 400 K simulations. Backbone ${\text{C}}_{\alpha }$ atoms were used for calculation. (A) RMSD. Coordinates at the first frame of each simulation were taken as the reference structure. (B) RMSF. Coordinate frames during the entire trajectory were used for calculation. The CD loop residues 41–45 have a high RMSF corresponding to the loop folding event at ∼155 ns (Figure 4C and Movie S1).
**Figure S2:** Total backbone Voronoi volume plotted over time.
**Figure S3:** Side‐chain volume expansion and fluctuation compared with solvent‐exposure. (A) Side‐chain volume expansion ($\langle V_{i}^{400\;\text{K}}\rangle -\langle V_{i}^{300\;\text{K}}$) plotted against the ratio of average solvent and protein neighbor counts at the two temperatures. Since the ratio of neighbor counts is not a robust measure of solvent exposure, plotting the volume increase against the changes in the ratio further blurs the correlation. (B) Side‐chain volume fluctuation at 300 and 400 K. Data in Figure 2E are plotted with color scale corresponding to average of the ratio between solvent and protein neighbor counts at respective temperatures as a measure of buried versus exposed residues. Lighter colors correspond to greater solvent exposure. All averages are for the first 180 ns.
**Figure S4:** Traces of all side‐chain volumes for both 300 and 400 K trajectories. Background colors of each residue label represent the log of solvent neighbor to protein neighbor ratios averaged over the 300 K simulation (color bar shown on bottom right), where a darker color indicates more buried side chains. Note that glycines are missing.
**Figure S5:** Traces of all backbone volumes for both 300 and 400 K trajectories. Plotting is done in the same way as for Figure S4. Data for glycines are added at the end that do not have side chains.
**Figure S6:** Degree of solvent exposure. (A) 300, (B) 400 K (all 200 ns were used for both simulations) Note that both the color scale and bar height are in logarithmic scale.

**Movie S1:** View of simulations at 300 and 400 K side by side. Notable events (such as loop folding and opening of β‐sandwich) are noted at the appropriate times throughout the trajectory.

## Data Availability

The data that support the findings of this study are openly available in the GitHub repository voro_dcd at https://github.com/sofiyabettencourt/voro_dcd.git.
