# Supplementary material for: Time‐Dependent Voronoi Analysis of Amino Acid Side‐Chain Packing in Simulations
Source: J Comput Chem. 2026 Sep 21;47(25):e70499. doi: 10.1002/jcc.70499 (PMC13594690; doi:10.1002/jcc.70499)
Supplement: Supplementary file 1 [file JCC-47-0-s001.pdf]

Supporting Information

Time-Dependent Voronoi Analysis of Amino Acid Side-Chain Packing in Simulations

Sofiya B. Bettencourt[1] and Wonmuk Hwang[1,2,3,4,⋆]

[1]Department of Biomedical Engineering, Texas A&M University, College Station, TX, USA
[2]Department of Materials Science & Engineering, Texas A&M University, College Station, TX, USA
[3]Department of Physics & Astronomy, Texas A&M University, College Station, TX, USA
[4]Center for AI and Natural Sciences, Korea Institute for Advanced Study, Seoul 02455, Republic of Korea
⋆Correspondence: hwm@tamu.edu

August 26, 2026

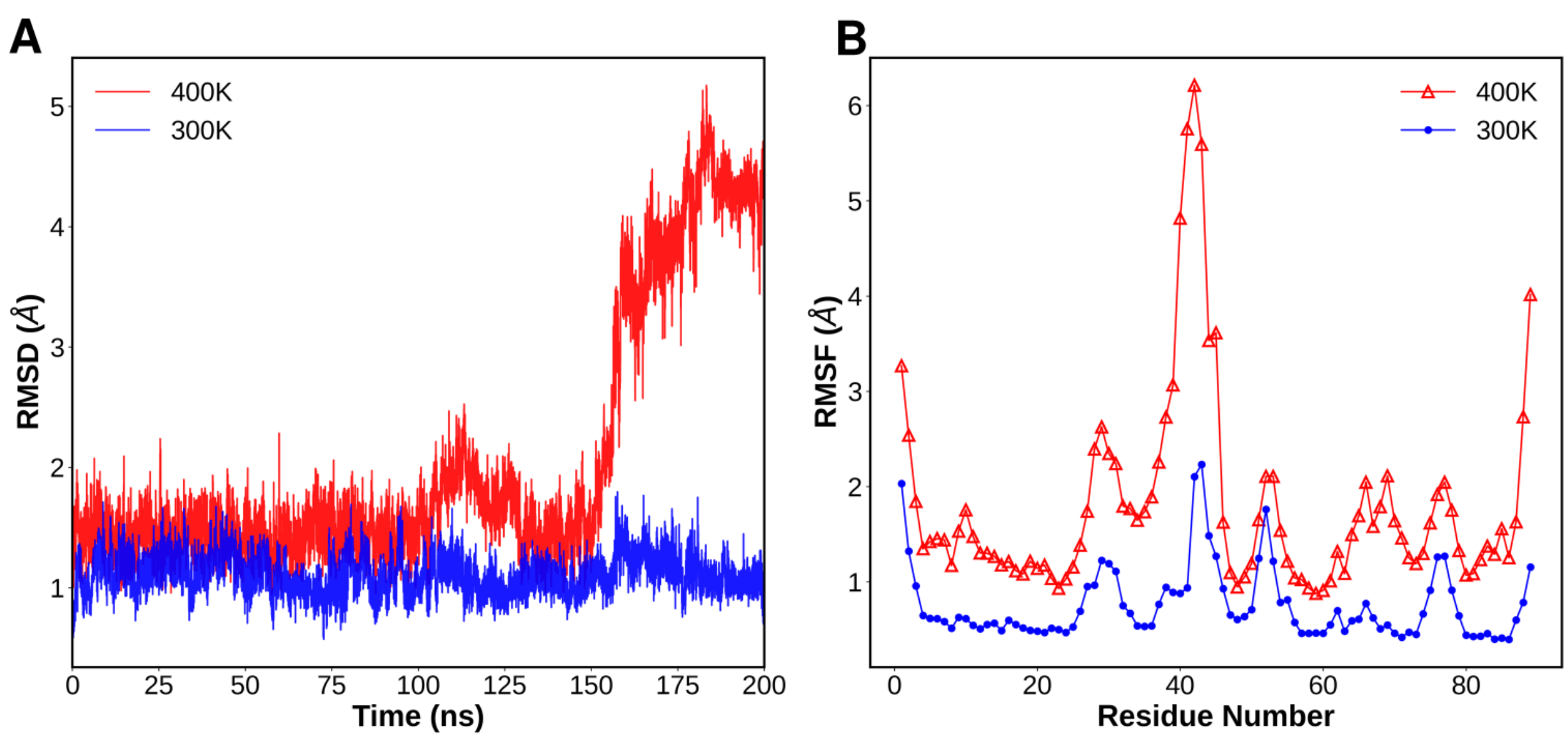

Figure S1: Conformational fluctuation during 300 K and 400 K simulations. Backbone C_α atoms were used for calculation. (A) RMSD. Coordinates at the first frame of each simulation were taken as the reference structure. (B) RMSF. Coordinate frames during the entire trajectory were used for calculation. The CD loop residues 41–45 have a high RMSF corresponding to the loop folding event at ∼155 ns (Fig. 4C and Movie S1).

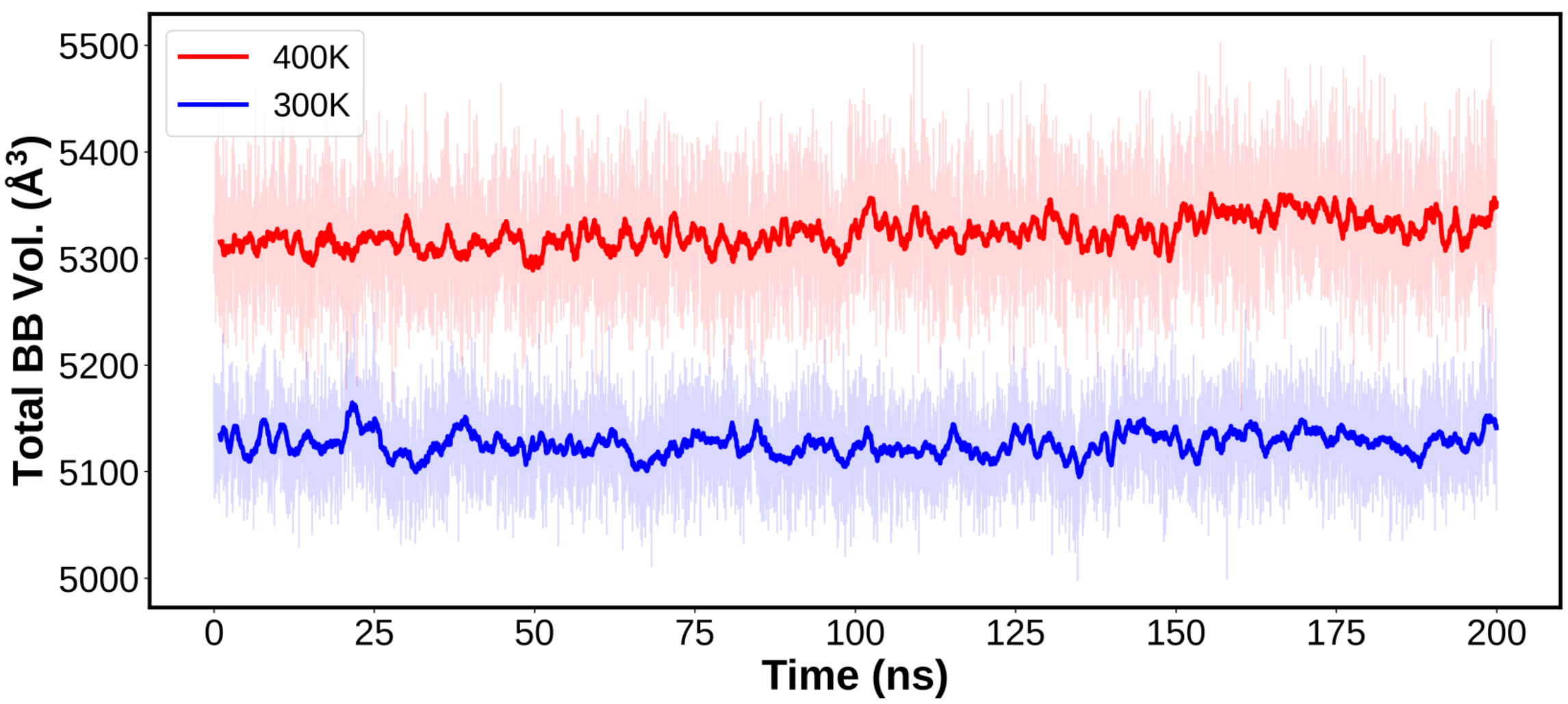

Figure S2: Total backbone Voronoi volume plotted over time.

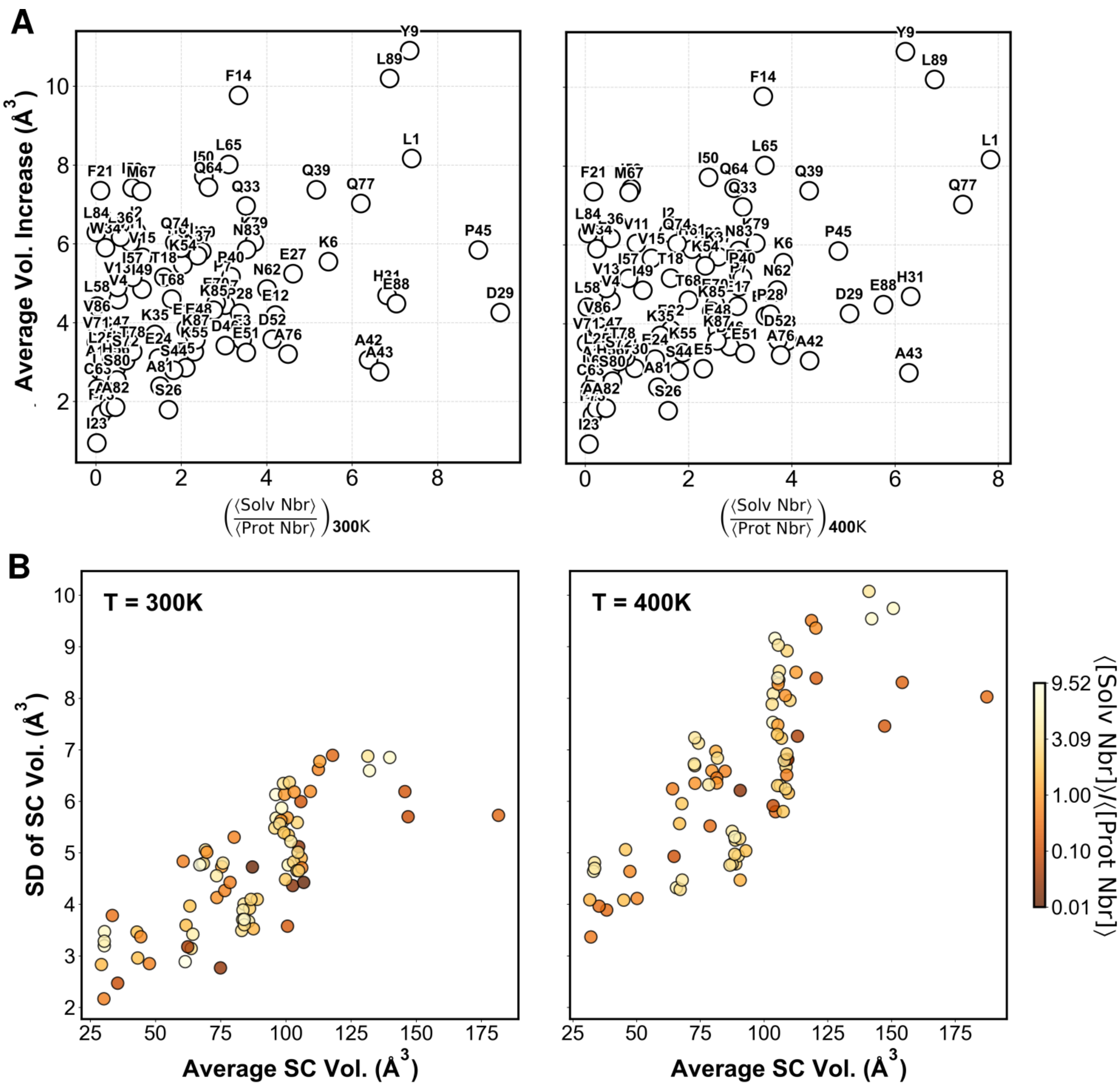

Figure S3: Side-chain volume expansion and fluctuation compared with solvent-exposure. (A) Side-chain volume expansion ($\langle V_i^{400\mathrm{K}} \rangle - \langle V_i^{300\mathrm{K}} \rangle$) plotted against the ratio of average solvent and protein neighbor counts at the two temperatures. Since the ratio of neighbor counts is not a robust measure of solvent exposure, plotting the volume increase against the changes in the ratio further blurs the correlation. (B) Side-chain volume fluctuation at 300 K and 400 K. Data in Fig. 2E are plotted with color scale corresponding to average of the ratio between solvent and protein neighbor counts at respective temperatures as a measure of buried vs. exposed residues. Lighter colors correspond to greater solvent exposure. All averages are for the first 180 ns.

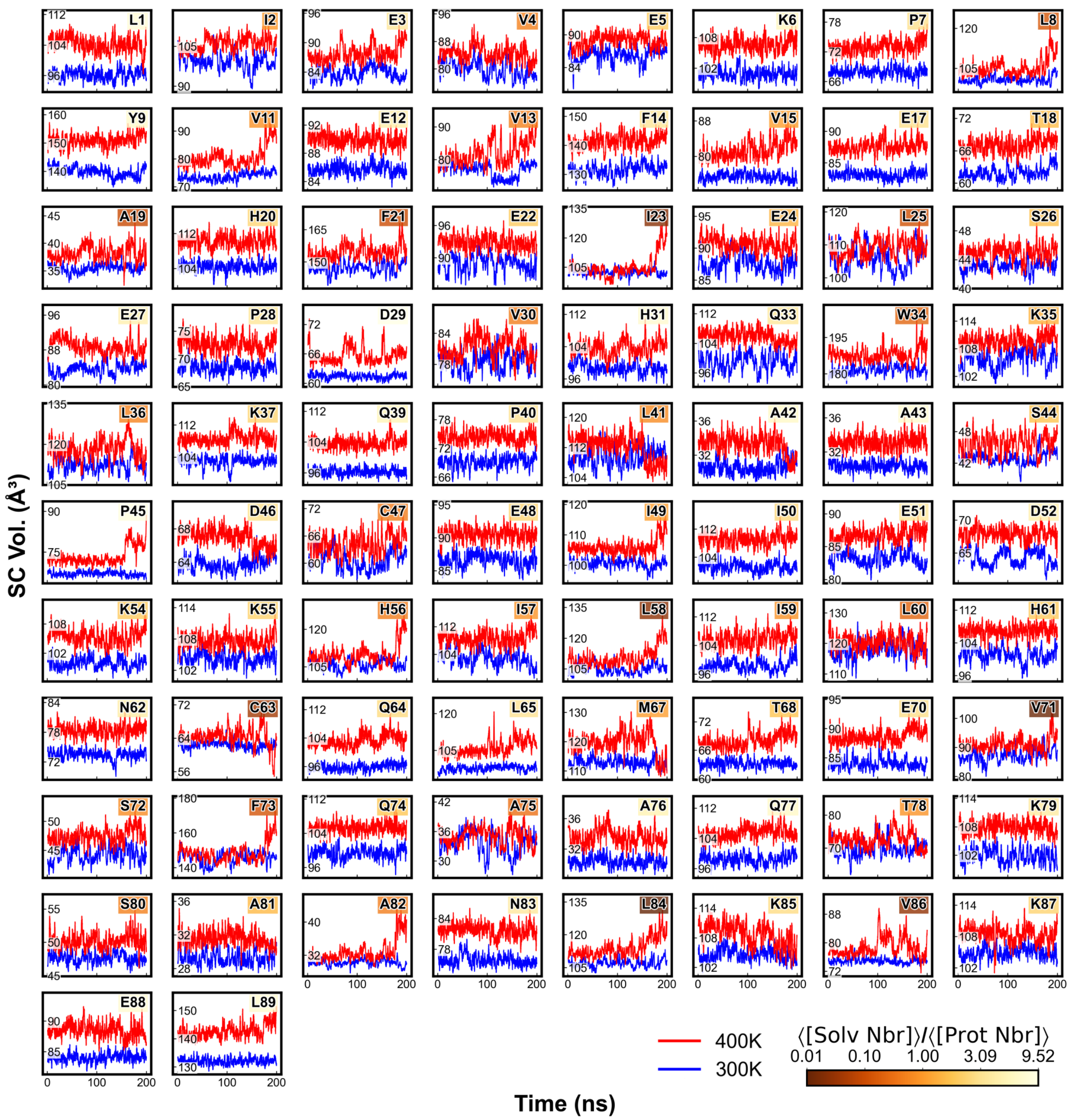

Figure S4: Traces of all side-chain volumes for both 300K and 400K trajectories. Background colors of each residue label represent the log of solvent neighbor to protein neighbor ratios averaged over the 300K simulation (color bar shown on bottom right), where a darker color indicates more buried side chains. Note that glycines are missing.

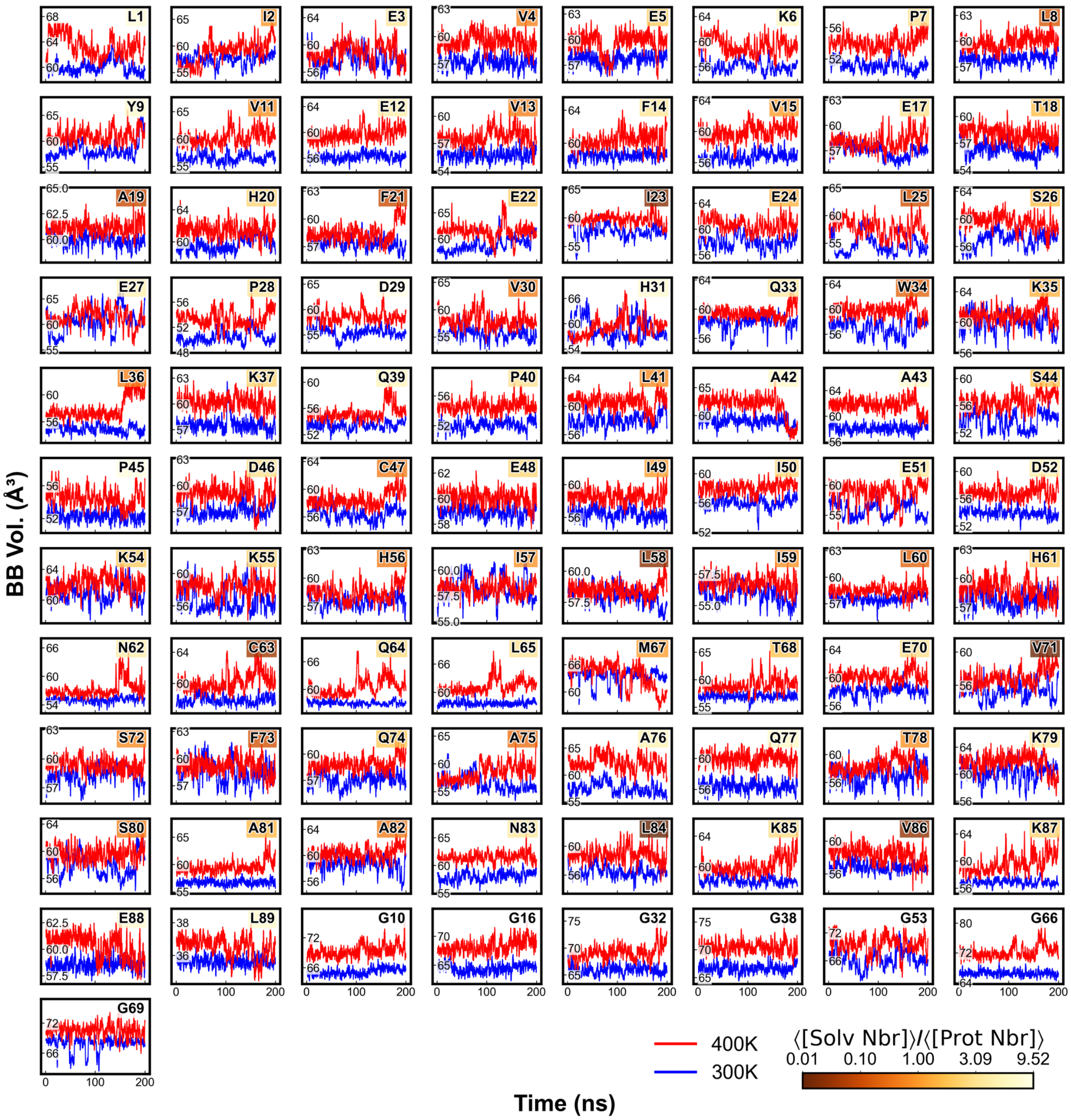

Figure S5: Traces of all backbone volumes for both 300K and 400K trajectories. Plotting is done in the same way as for Fig. S4. Data for glycines are added at the end that do not have side chains.

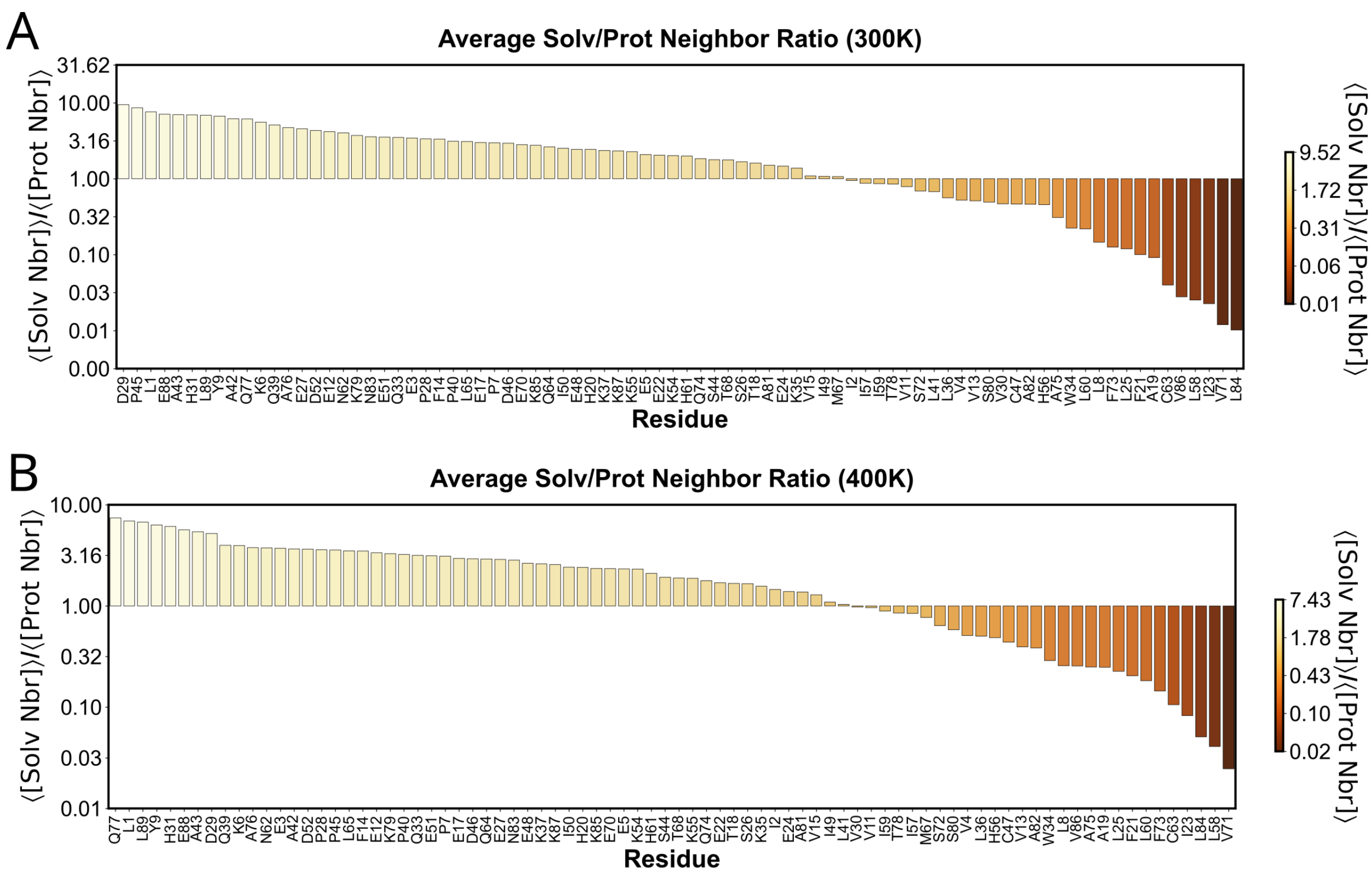

Figure S6: Degree of solvent exposure. (A) 300 K, (B) 400 K (all 200 ns were used for both simulations) Note that both the color scale and bar height are in logarithmic scale.

Movie S1. View of simulations at 300 K and 400 K side by side. Notable events (such as loop folding and opening of β-sandwich) are noted at the appropriate times throughout the trajectory.
